# Impact of Tetracycline Stress on Water Quality and Rhizosphere Microbial Communities of *Eichhornia crassipes*: Implications for Bioremediation

**DOI:** 10.3390/microorganisms13040893

**Published:** 2025-04-13

**Authors:** Dandan Sun, Huan Zhang, Guojun Pan, Zhenhua Zhang, Jincheng Xing, Jiangye Li, Yan Gao, Wei Chen, Xin Lu

**Affiliations:** 1 Key Laboratory for Saline-Alkali Soil Improvement and Utilization, Ministry of Agriculture and Rural Affairs/Institute of Agricultural Resources and Environment, Jiangsu Academy of Agricultural Sciences, Nanjing 210014, China; dandan_sun@hotmail.com (D.S.); zhanghuan2025@126.com (H.Z.); pangj202412@163.com (G.P.); zhenhuaz70@hotmail.com (Z.Z.); jiangye1232@jaas.ac.cn (J.L.); ygao@jaas.ac.cn (Y.G.); wchen@jaas.ac.cn (W.C.); 2 School of the Environment and Safety Engineering, Jiangsu University, Zhenjiang 212013, China; 3 Institute of Jiangsu Coastal Agricultural Sciences, Yancheng 224002, China; sdauxxx@163.com; 4 School of Agriculture and Environment, The University of Western Australia, Crawley, WA 6009, Australia

**Keywords:** antibiotics, bioremediation, rhizospheric microbiota, water pollution, aquatic plant

## Abstract

To examine the impact of antibiotic contamination on water quality and rhizospheric microbial communities, a simulated cultivation experiment was employed to investigate the potential impacts of tetracycline (Tet) stress on water quality and microbial community composition in the rhizosphere of *Eichhornia crassipes* (*E. crassipes*), with a focus on its implications for bioremediation strategies. The results showed a significant disruption in microbial diversity and community structure in the rhizosphere at varying accumulated Tet concentrations (0, 2, 5, and 10 mg·L^−1^). The microbial communities displayed resilience and functional stability from the low (2 mg·L^−1^) to moderate (5 mg·L^−1^) accumulated Tet concentrations; while significant root decay and a marked decline in microbial diversity were observed at the high (10 mg·L^−1^) accumulated Tet concentration. Some bacterial taxa, including Rhizobiaceae (0.34%), Comamonadaceae (0.37%), and Chitinophagaceae (0.38%), exhibited notable enrichment under Tet stress, underscoring their functional roles in nitrogen cycling, organic matter decomposition, and antibiotic degradation. Physicochemical changes in the rhizosphere, such as shifts in low-molecular-weight organic acids (LMWOAs), nutrient cycling, and total organic carbon (TOC), revealed Tet-induced metabolic adaptations and environmental alterations. Correlation analysis between environmental factors and dominant operational taxonomic units (OTUs) highlighted the putative intricate interplay between microbial activity and Tet stress. These findings underscore the dual impact of Tet as both a stressor and a selective agent, favoring antibiotic-resistant taxa while suppressing sensitive groups. This study provides foundational insights into the ecological and functional dynamics of microbial communities under antibiotic contamination conditions and highlights the potential of rhizospheric microbial communities in the rhizosphere for bioremediation in Tet-polluted ecosystems.

## 1. Introduction

Tetracycline (Tet), a widely used broad-spectrum antibiotic, has become a ubiquitous environmental pollutant in aquatic ecosystems due to excessive application and improper disposal. Its environmental persistence poses significant ecological risks, including the proliferation of microbial-borne antibiotic resistance genes (ARGs) and disruptions to microbial community structures [1,2,3,4]. Tet degradation in the environment involves complex interactions among physicochemical conditions, organic and inorganic constituents, and microbial activities. Abiotic processes such as hydrolysis [5], photodegradation [6], and advanced oxidation reactions [7] can contribute to Tet breakdown. However, these processes exhibit slow and incomplete degradation rates due to limited efficacy in specific environmental conditions (e.g., turbid aquatic systems), resulting in persistent Tet residues. This makes them insufficient to fully mitigate environmental impact, particularly in highly polluted water [8].

Microbial degradation, in contrast, has emerged as a promising and sustainable approach. Specific bacterial taxa, such as *Pseudomonas fluorescens*, have demonstrated the capacity to degrade Tet efficiently, providing critical insights into bioremediation strategies [9]. *Pseudomonas fluorescens*, being a facultative anaerobe, can utilize oxygen in aerobic conditions and switch to alternative electron acceptors such as nitrates or sulfates in anaerobic environments, which may also play a role in Tet degradation under low oxygen conditions [9]. The microbial degradation of Tet in aquatic environments requires microbial habitats and carbon and nitrogen sources [10]. In addition, the appropriate pH value and oxygen content have a significant impact on the degradation capability of microorganisms [11,12]. Phytoremediation involving plant–microorganism combined bioremediation of antibiotic contaminated water has gradually attracted attention because aquatic plant roots are usually suspended in water, which can provide appropriate habitats and release root exudates as carbon and nitrogen sources for microbes. In addition, plants can produce oxygen through photosynthesis. This oxygen is then transported to the roots to create a favorable oxygen-rich condition for rhizosphere-attached microorganisms to degrade and transform pollutants [13,14,15].

*Eichhornia crassipes* (water hyacinth, Liliopsida: Pontederiaceae), a commonly used aquatic phytoremediation plant, plays a critical role in pollutant remediation by altering the physicochemical properties of water and interacting with microbial communities in the rhizosphere [16]. The rhizosphere is a dynamic microenvironment characterized by complex plant–microbe interactions, high microbial diversity and enriched functional potential, including ARG-carrying taxa and xenobiotic-degrading enzymes [17]. Multiple studies have demonstrated that *E. crassipes* exhibits significant effectiveness in the removal of antibiotic pollutants [16,18,19,20]. Through mechanisms such as adsorption, plant uptake, and microbial degradation, *E. crassipes* efficiently removes various antibiotics from water bodies, including ciprofloxacin [18,21], tetracycline [16], and sulfonamides [19]. At different growth stages, *E. crassipes* exhibits varying characteristics in antibiotic absorption and metabolism, with the seedling stage showing enhanced drug resistance and migration capacity, making it suitable for long-term drug degradation [21]. *E. crassipes* also enhances the functionality of remediation systems through synergistic interactions with microbial communities, offering effective solutions for phytoremediation of antibiotic-contaminated water [20].

Despite these advances, the mechanisms governing Tet degradation and dynamics of rhizospheric microbial community during phytoremediation remain poorly understood. In particular, the interaction among Tet stress, microbial activities and environmental factors such as pH, organic carbon, and nitrogen availability has received limited attention. Microbial consortia responsible for efficient Tet degradation have recently been enriched, demonstrating effective Tet degradation across a wide pH range (4–10) and under optimal culture conditions [22]. Understanding the interactions mentioned above during phytoremediation is essential for optimizing bioremediation strategies and minimizing the ecological risks associated with antibiotic contamination.

The existing studies have demonstrated that Tet stress selectively enriched the phylum Acidobacteria and the genera such as Hyphomicrobium, Bryobacter, and Pseudomonas in Tet-contaminated environments [23]. Although few studies have focused on the change in microbial community and gene mutation in the rhizosphere of aquatic plants under Tet stress, related studies have shown that the increased abundance of RND efflux pump system genes *acrB* and *mexB* strongly correlated to Pseudomonas dominance in activated sludge systems exposed to Tet at the high selective pressures (>5 mg/L). Metagenomic analyses further confirmed that Tet stress drives microbial community restructuring through dose-dependent selection, with Pseudomonas attaining resistance via sustained expression of the *acrAB-tolC* operon, which is supported by elevated gene copy numbers in Tet-treated systems [24]. In this study, a simulated cultivation experiment was employed to investigate the impacts of Tet stress on water quality and microbial community composition in the rhizosphere of *E. crassipes*. The objectives of this study were to (1) identify key microbial taxa enriched exposed to Tet; (2) elucidate the relationship between environmental factors and microbial dynamics; and (3) explore the potential of plant–microbe systems for sustainable bioremediation applications.

## 2. Materials and Methods

### 2.1. Preparation of Experimental Materials

The *E. crassipes* used in the experiment was purchased from Jingzhou Horticulture Co., Ltd. in Suqian, Jiangsu, China. After pre-cultivation in pond water (from a pond used for surrounding domestic sewage discharge) in Jiangsu Academy of Agricultural Sciences for two weeks, plants with relatively consistent morphology and physiological activity (similar in height with a difference within 2 mm and similar in weight with a difference within 5 g) were selected for subsequent studies. Precisely 0.2 g of tetracycline (Shanghai Macklin Biochemical Co., Ltd., Shanghai, China, CAS: 60-54-8, Lot#: C15403025) was dissolved in 50 mL of anhydrous ethanol (Sinopharm Chemical Reagent Co., Ltd., Shanghai, China, purity 99.7%, CAS: 64-17-5) to prepare a 4000 mg L^−1^ Tet stock solution for later use.

### 2.2. Tet Stress of E. crassipes

The simulated cultivation system consisted of six 12 L-polyethylene containers, each planted with 10 plants and filled with 10 L of pre-treated pond water. The experiment included a control group (CK, without Tet) and a Tet exposure group (Tet treatment), and tests for each group were conducted in triplicate. A gradient-increasing dosing mode was adopted for Tet treatment: On 26 August 2024 (Day 0), Tet was initially accumulated to 2 mg·L^−1^ on Day 3. Subsequently, the concentration was gradually increased by regularly supplementing the 4000 mg L^−1^ stock solution and accumulated to approximately 5 mg·L^−1^ and 10 mg·L^−1^ on Day 9 and Day 10 respectively, based on our previous study [16]. This design simulated a composite stress scenario of continuous input of antibiotic pollution in water and long-term biological exposure, as employed by Zhang et al., to study the effects of Tet on microbial communities and ARG levels in aerobic granular sludge in a sequencing batch reactor, as employed by Zhang et al. [25].

Sampling nomenclature followed a specific convention as follows: W and R in the sample name represent water samples (W-Tet1, W-Tet2, W-Tet3, W-CK1, W-CK2, and W-CK3) and root samples (R-Tet1, R-Tet2, R-Tet3, R-CK1, R-CK2, and R-CK3), respectively. Tet1, Tet2, and Tet3 represent Tet treatments with the addition of accumulated concentrations of 2 mg·L^−1^, 5 mg·L^−1^, and 10 mg·L^−1^ at respective sampling time points; CK1, CK2, and CK3 are control groups corresponding to the treatments.

System sampling was performed three times on Days 3, 9, and 19, corresponding with accumulated Tet concentrations of 2, 5, and 10 mg L^−1^, respectively, including water samples of the *E. crassipes* rhizosphere for analyzing physicochemical properties (such as pH, ammonium nitrogen (NH_4_^+^-N), nitrate nitrogen (NO_3_^−^-N), total nitrogen (TN), and total organic carbon (TOC)), microbial communities in water, and root samples for 16s rDNA sequencing analysis. At the end of the experiment, an additional rhizospheric water sample was collected for root exudate measurement. *E. crassipes* was harvested, rinsed with deionized water, and drained, and morphological parameters were recorded on Day 19.

### 2.3. Physicochemical Analysis

The physicochemical parameters of water quality, including pH, ammonium nitrogen (NH_4_^+^-N), nitrate nitrogen (NO_3_^−^-N), total nitrogen (TN), and total organic carbon (TOC) were analyzed in strict accordance with the Methods for Monitoring and Analysis of Water and Wastewater (4th Edition) [26]. The pH measurements were performed using a pH meter (PHSJ-3F laboratory, Shanghai Leici Instrument Factory, Shanghai, China). NH_4_^+^-N and NO_3_^−^-N concentrations were simultaneously determined through flow injection analysis coupled with salicylic acid spectrophotometry using a continuous flow analyzer (SKALAR san++, Skalar, Breda, The Netherlands). TOC quantification was conducted via high-temperature catalytic combustion oxidation (850 °C) employing a TOC analyzer (multi N/C 2100S, Analytik Jena Instrument (Beijing) Co., Ltd., Beijing, China). TN was measured through alkaline potassium persulfate digestion followed by UV spectrophotometric detection at 220 nm using a UV–Vis spectrophotometer (TU-1080, Beijing Purkinje General Instrument Co., Ltd., Beijing, China).

### 2.4. Biomass and Root Exudates

For biomass quantification, plants were carefully removed from containers, surface moisture was blotted, then total dry weight, root length, and plant height were recorded. The root length and plant height were measured by selecting the 3 tallest *E*. *crassipes* plants in each bucket. Subsequently, all ten plants in each bucket were placed into paper bags and subjected to drying in an electric thermostatic drying oven (DHG-9240A, Jing Hong Laboratory Instrument Co., Ltd., Shanghai, China). Initially, the temperature was set to 100–105 °C for 10 min then lowered to approximately 70–80 °C to allow plants to dry until a constant weight was achieved. The samples were then removed from the oven, cooled to room temperature in a desiccator, and weighed.

Root exudate collection was performed by sampling 300 mL of hydroponic solution from each bucket. Each sample underwent filtration through 0.45 μm syringe filters followed by lyophilization using a freeze dryer (FD-1A-50, BiLon Instrument Co., Ltd., Shanghai, China), with resultant samples stored at −20 °C for subsequent High-Performance Liquid Chromatography (HPLC, Waters, Milford, CT, USA) analysis.

HPLC Configuration: Chromatographic separation was achieved using a reversed-phase C18 anion-exchange column (Agilent Zorbax-Aq, 4.6 × 250 mm, 5 μm, Agilent Technologies, Inc., Waldbronn, Germany). The freeze-dried samples were dissolved by a 5 mL mobile phase. Operational parameters included the following: 10 μL injection volume, 30 °C column temperature, and a mobile phase comprising 35% ultrapure water, 2% methanol, and 63% NaH_2_PO_4_ solution (1.0 mM, pH 2.8) at 1 mL min^−1^ flow rate. Organic acids in root exudates were identified by comparing retention times with eight reference standards: tartaric, formic, malic, propionic, acetic, citric, succinic, and lactic acids. Quantification was established through five-point calibration curves (R^2^ > 0.995) correlating peak areas with analyte concentrations [27].

### 2.5. Rhizosphere Microbial Sampling and Sequencing

Rhizosphere microbial sampling was initiated by collecting 20 g root segments using flame-sterilized scissors. Surface-adhered moisture was removed via sterile filter paper blotting before flash-freezing in liquid nitrogen. Concurrently, 500 mL rhizospheric water samples were vacuum-filtered through 0.22 μm Polyvinylidene Fluoride (PVDF) membranes for microbial enrichment. Both root and membrane samples were cryopreserved at −80 °C and transported to MAGIGENE Biotechnology Co., Ltd. (Shenzhen, China) for downstream processing.

Genomic DNA extraction was performed using the E.Z.N.A.^®^ Soil DNA Kit (Omega Bio-tek, Norcross, GA, USA), with DNA integrity verified through A_260_/A_280_ ratios (1.8–2.0) measured by a NanoDrop One spectrophotometer (Thermo Fisher Scientific, Waltham, MA, USA). Bacterial 16S rRNA gene amplification targeted the V3-V4 hypervariable regions using universal primers 338F (5′-ACTCCTACGGGAGGCAGCAG-3′) and 806R (5′-GGACTACHVGGGTWTCTAAT-3′) [28]. Purified amplicons underwent paired-end sequencing (2 × 300 bp) on an Illumina MiSeq platform (Illumina Inc., San Diego, CA, USA).

Sequencing data preprocessing involved the following: (1) raw read quality control using the FASTQ toolkit [29] with the Q20 threshold; (2) paired-end sequence merging via FLASH v1.2.11 [30], allowing 10% mismatch tolerance; (3) generation of high-fidelity clean tags through chimera removal using the UCHIME algorithm.

### 2.6. Data Analysis

High-quality sequences were clustered into operational taxonomic units (OTUs) at a 97% similarity threshold using Uparse (v11.0.667) [31]. Sequence read counts were normalized through cumulative sum scaling (CSS) using the DESeq2 package (v1.40.2) to mitigate sequencing depth heterogeneity while preserving ecological signals [31,32]. Representative sequences of each OTU were taxonomically annotated using the RDP Classifier against the Silva v138 database [33,34].

Alpha diversity metrics, including Shannon and Simpson indices, were calculated using Mothur software (v1.48.1) [35] to evaluate microbial diversity within samples. Bray–Curtis distances were computed from OTU abundance tables, and Principal Coordinate Analysis (PCoA) was performed using the vegan package in R (v2.2-1) [36]. Microbial community composition was visualized through bar plots of relative abundances for dominant bacterial taxa, generated using the ggplot2 package (v3.5.1) [37] in R. Sample clustering heatmaps based on Bray–Curtis distances were created with the heatmap package (v1.0.12) [38]. Correlations between dominant taxa and environmental factors (e.g., pH, NH_4_^+^-N, NO_3_^−^-N, TN, TOC) were analyzed using Spearman correlation (v4.4.0) coefficients and visualized with the corrplot package (v0.95) [39].

Statistical analyses were conducted using SPSS 27.0 (IBM Corp., Armonk, NY, USA), and data visualization was performed with Origin 2025 (© OriginLab Corporation, Northampton, MA, USA).

## 3. Results

### 3.1. Changes in E. crassipes Growth and Physicochemical Indicators in Rhizospheric Water Under Tet Stress

#### 3.1.1. Growth of *E. crassipes* Under Tet Stress

**Figure 1:**
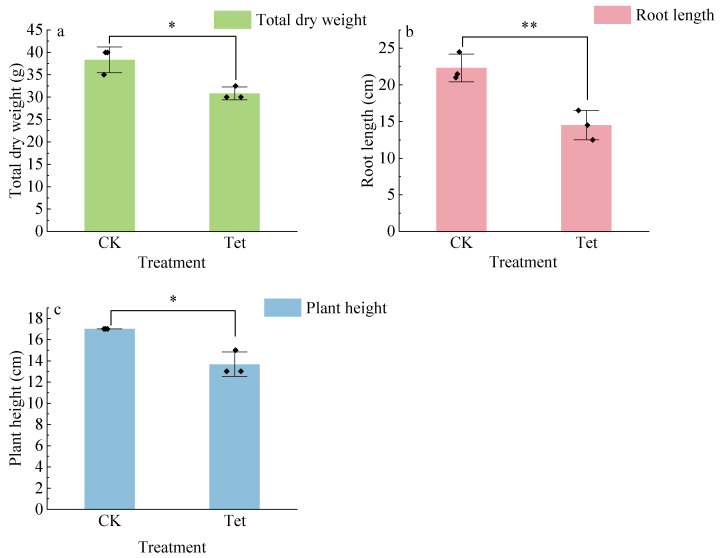
Total dry weight (**a**), root length (**b**), and plant height (**c**) of *E. crassipes* under Tet stress on Day 19. CK represents the control group (no Tet), and Tet corresponds to the accumulated tetracycline treatment. Data are presented as mean ± SD (*n* = 3). Asterisks indicate significant differences, with a single asterisk (**a**,**c**) representing the Mann–Whitney U test (*p* < 0.05), and double asterisks (**b**) denote the *t*-test (*p* < 0.01).

The dry weight, root length, and plant height of *E. crassipes* were significantly affected by Tet stress. As shown in Figure 1, the total dry weight was significantly lower in Tet treatment than the CK. Compared to the CK, root length and plant height in Tet treatment significantly decreased by 58.5% and 32.3%, respectively.

#### 3.1.2. Changes in pH in the Rhizospheric Water

**Figure 2:**
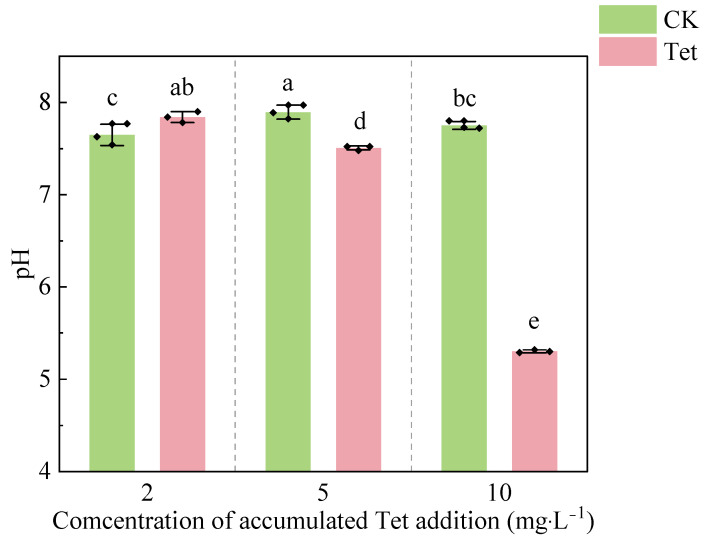
The pH in the rhizospheric water of *E. crassipes* on Days 3, 9, and 19, corresponding with accumulated Tet concentrations of 2, 5, and 10 mg L^−1^, respectively. CK represents the control group (no Tet), and Tet corresponds to the accumulated tetracycline treatment. Data are presented as mean ± SD (*n* = 3). Different lowercase letters indicate significant differences (*p* < 0.05) based on Duncan’s multiple range test.

Compared to the CK, the pH in the rhizospheric water gradually decreased in Tet treatment (Figure 2). The pH in the CK stayed relatively stable, while a significant decline was observed in Tet treatment. When the accumulated Tet concentration reached 10 mg·L^−1^, the pH dropped to 5.30, which was significantly lower than that of the CK (pH = 7.76). The sign of root decay was observed when the accumulated Tet concentration reached 10 mg·L^−1^ during the experiment.

#### 3.1.3. Changes in NH_4_^+^-N, NO_3_^−^-N and TN in the Rhizospheric Water

**Figure 3:**
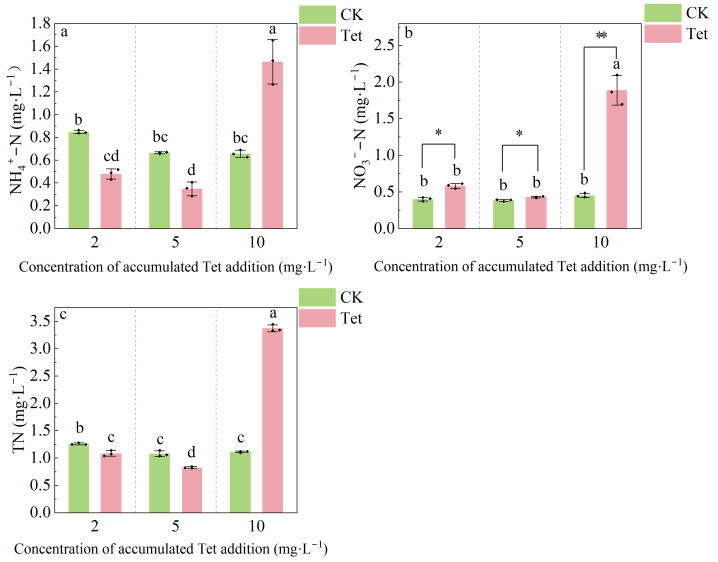
Changes in (**a**) NH_4_^+^-N, (**b**) NO_3_^−^-N, and (**c**) TN concentrations in the rhizospheric water of *E. crassipes* on Days 3, 9, and 19, corresponding with accumulated Tet concentrations of 2, 5, and 10 mg L^−1^, respectively. CK represents the control group (no Tet), and Tet corresponds to the accumulated tetracycline treatment. Data are presented as mean ± SD (*n* = 3). Different lowercase letters indicate significant differences (*p* < 0.05) based on Duncan’s multiple range test. Asterisks indicate the significant difference between the CK and Tet treatment at the same sampling time point based on the *t*-test, with a single asterisk representing *p* < 0.05 and double asterisks representing *p* < 0.01.

As shown in Figure 3, the concentrations of NH_4_^+^-N, NO_3_^−^-N, and TN in the CK remained relatively stable. In contrast, NH_4_^+^-N, NO_3_^−^-N, and TN levels initially decreased in Tet treatment then sharply increased as accumulated Tet addition increased. The concentrations of NH_4_^+^-N, NO_3_^−^-N, and TN declined when the amount of Tet concentration accumulated from 2 mg·L^−1^ to 5 mg·L^−1^. However, the concentrations of NH_4_^+^-N, NO_3_^−^-N, and TN significantly increased upon a further increase in Tet concentration to 10 mg·L^−1^.

#### 3.1.4. Changes in TOC in the Rhizospheric Water

**Figure 4:**
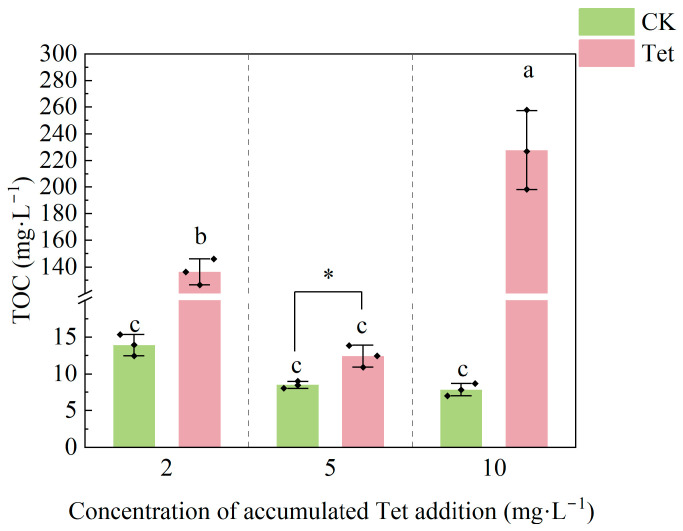
Total organic carbon (TOC) in the rhizospheric water of *E. crassipes* on Days 3, 9, and 19, corresponding with accumulated Tet concentrations of 2, 5, and 10 mg L^−1^, respectively. CK represents the control group (no Tet), and Tet corresponds to the accumulated tetracycline treatment. Data are presented as mean ± SD (*n* = 3). Different lowercase letters indicate significant differences (*p* < 0.05) based on Duncan’s multiple range test. Asterisks indicate the significant difference between the CK and Tet treatment at the same sampling time point (the amount of accumulated Tet was 5 mg·L^−1^), with a single asterisk representing *p* < 0.05 based on the *t*-test.

As shown in Figure 4, the TOC concentration significantly fluctuated under Tet stress. When the accumulated Tet concentration increased from 2 to 5 mg·L^−1^, the TOC concentration suddenly dropped by one order of magnitude. However, when the accumulated Tet concentration increased from 5 to 10 mg·L^−1^, the TOC concentration significantly increased, which was accompanied by a sign of root decay, leading to a substantial rise in the TOC concentration in the rhizospheric water. In contrast, the TOC concentration in the CK showed a sign of decreasing trend, while no significant difference was observed.

#### 3.1.5. Changes in LMWOAs in the Rhizospheric Water

**Figure 5:**
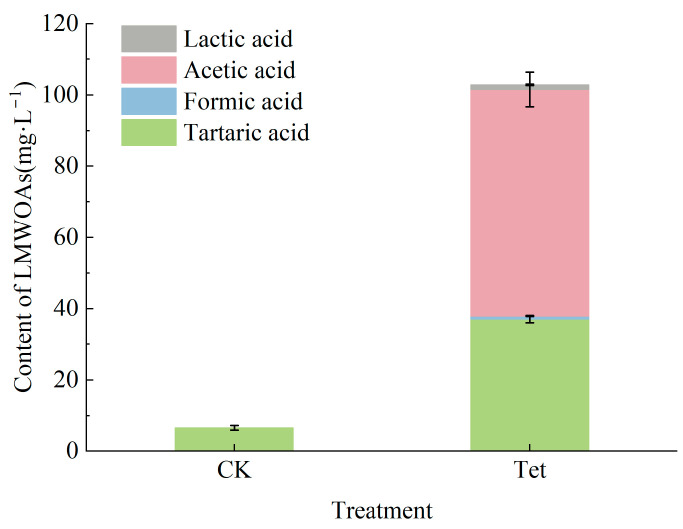
The contents of low-molecular-weight organic acids (LMWOAs) in the rhizospheric water of *E. crassipes* under Tet stress on Day 19. CK represents the control group (no Tet), and Tet corresponds to the accumulated tetracycline treatment. Data are presented as mean ± SD (n = 3).

Under Tet stress, the composition and concentration of low-molecular-weight organic acids (LMWOAs) in the rhizospheric water changed significantly (*p* < 0.05), as shown in Figure 5. The tartaric acid was the only detected organic acid in the CK, with a concentration of 6.52 ± 0.68 mg·L^−1^. In contrast, four types of LMWOAs, namely, tartaric acid, formic acid, acetic acid, and lactic acid, were detected in Tet treatment, with concentrations of 36.99 ± 1.00, 0.90 ± 0.13, 63.62 ± 4.87, and 1.35 ± 0.21 mg·L^−1^, respectively, leading to a significant increase in the total amount of organic acids. The concentration of tartaric acid under Tet treatment conditions increased significantly compared to the CK. The acetic acid showed the highest concentration among all LMWOAs detected in the present study.

### 3.2. Changes in Microbial Communities in the Root and Rhizospheric Water of E. crassipes Under Tet Stress

Illumina MiSeq sequencing generated 702,592 high-quality bacterial 16S rRNA gene reads after stringent quality control. Of these, 419,209 reads originated from water samples, while 283,383 were derived from rhizospheric microbial communities. Rarefaction and normalization procedures identified 5864 operational taxonomic units (OTUs) at a 97% similarity threshold. The raw sequencing data are available in the NCBI Sequence Read Archive (SRA) under BioProject accession number PRJNA1244745. This comprehensive dataset enabled detailed analyses of microbial community composition and diversity in response to environmental conditions and Tet exposure.

#### 3.2.1. Principal Coordinate Analysis

**Figure 6:**
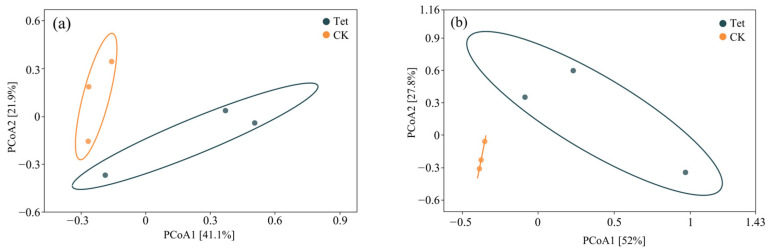
PCoA of microbial community composition based on the Bray–Curtis distance matrix. (**a**) Water microbial communities, (**b**) rhizospheric microbial communities under Tet stress and in the CK. CK represents the control group (no Tet), and Tet corresponds to the accumulated tetracycline treatment.

The PCoA revealed divergent microbial community structures between Tet-stressed and control conditions in both aquatic and rhizospheric environments (Figure 6). In water samples (Figure 6a), PCoA1 and PCoA2 explained 41.1% and 21.9% of the variation, respectively, with water communities displaying greater variability, reflecting the dynamic abiotic factors in aquatic ecosystems. Root samples (Figure 6b) exhibited tighter clustering, indicating a stronger influence of root-associated factors. Tet-treated samples showed greater dispersion in PCoA plots across both environments, suggesting shifts toward communities enriched in antibiotic-resistant taxa. These findings underscore the strong impacts of environmental conditions and Tet stress on microbial community composition.

#### 3.2.2. Relative Abundances at the Phylum Level

**Figure 7:**
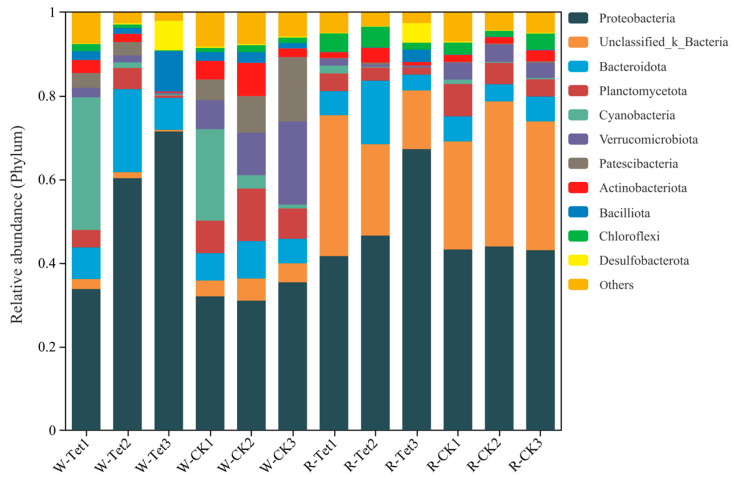
Relative abundance of dominant microbial taxa at the phylum level in water (W) and root (R) samples under control (CK) and Tet treatment conditions on Days 3, 9, and 19, corresponding with Tet accumulated concentrations of 2, 5, and 10 mg L^−1^, respectively. W-Tet1, W-Tet2, W-Tet3, W-CK1, W-CK2, and W-CK3 represent water samples, and R-Tet1, R-Tet2, R-Tet3, R-CK1, R-CK2, and R-CK3 represent root samples. CK represents the control group (no Tet), and Tet corresponds to the accumulated tetracycline treatment. The vertical axis represents the relative abundance of each major microbial community at the phylum level.

At the phylum level, the relative abundances of dominant microbial taxa in each sample are presented in Figure 7. Proteobacteria (46.6%) emerged as the most dominant phylum across all samples, underscoring its ecological versatility and resilience. In water samples, secondary phyla such as Bacteroidota (8.25%) and Actinobacteriota (2.69%) exhibited notable increases in relative abundance under Tet stress, likely due to their roles in organic matter decomposition and adaptive resistance mechanisms. Water samples of Tet treatment showed elevated abundances of these taxa, while the CK maintained relatively stable community compositions, further emphasizing the selective influence of Tet exposure.

In root samples, Proteobacteria (46.6%) remained the dominant phylum, while Actinobacteriota (2.69%) and Bacilliota (2.47%) were significantly enriched under Tet stress, suggesting their involvement in antibiotic degradation and stress resilience. Sensitive taxa, such as Verrucomicrobiota (8.25%) and Planctomycetota (5.42%), were suppressed when exposed to Tet. The rhizospheric microenvironment, expectedly shaped by root exudates and localized nutrient gradients, intensified these selective pressures, leading to more pronounced shifts compared to water rhizospheric samples.

#### 3.2.3. Microbial Community Shifts Under Tet Stress

The dominant microbial taxa and their ecological significance were analyzed at 2 mg·L^−1^, 5 mg·L^−1^, and 10 mg·L^−1^ of accumulated Tet addition. The application of 10 mg·L^−1^ accumulated Tet addition resulted in extensive root decay, likely causing deviations in microbial community characteristics from the plant’s normal physiological state. Therefore, this study primarily focuses on the dynamic changes in microbial communities with the low to moderate accumulated addition of Tet (2 to 5 mg·L^−1^).

##### Water Sample Analysis

**Figure 8:**
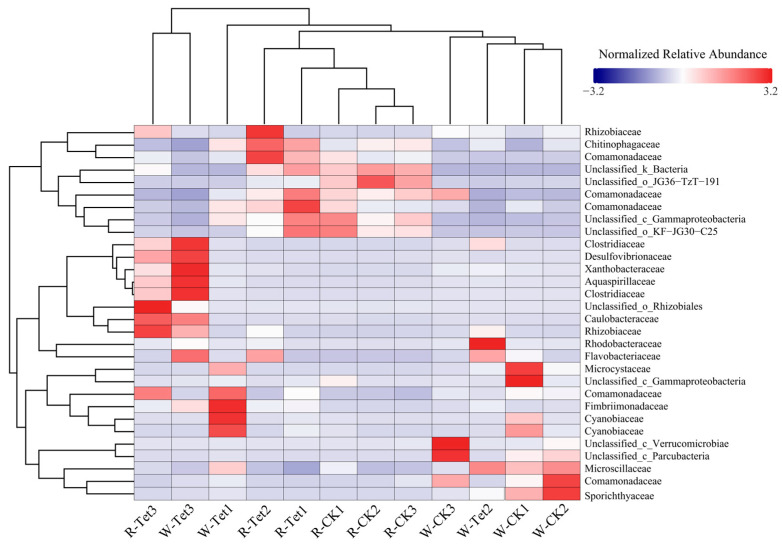
Heatmap of genus-level abundance clustering for the 30 most relatively abundant species. Samples were collected on Days 3, 9, and 19, corresponding with accumulated Tet concentrations of 2, 5, and 10 mg L^−1^, respectively. W-Tet1, W-Tet2, W-Tet3, W-CK1, W-CK2, and W-CK3 represent water samples, and R-Tet1, R-Tet2, R-Tet3, R-CK1, R-CK2, and R-CK3 represent root samples. CK represents the control group (no Tet), and Tet corresponds to the accumulated tetracycline treatment. The clustering tree on the left side of the figure is the species clustering tree; the clustering tree above is the sample clustering tree, reflecting the similarity of community composition between samples. The values corresponding to the middle squares are the Z-values obtained after standardizing the relative abundance of each row of species; that is, the Z-value of a sample in a certain classification is the difference between the relative abundance of the sample in that classification and the average relative abundance of all samples in that classification divided by the standard deviation of all samples in that classification. The redder the color of the square, the higher the relative abundance of the phylum among the samples, meaning only horizontal comparison is possible and not vertical.

The clustering and species abundance patterns in water samples (with W-CK1 and W-CK2 combined as the CK and W-Tet1 and W-Tet2 denoting accumulated Tet addition treatment of 2 and 5 mg·L^−1^, respectively) demonstrated significant shifts in microbial community structure under Tet stress (Figure 8). Rhodobacteraceae (genus *Rhodobacter*, 3.71%) showed substantial enrichment at 5 mg·L^−1^ accumulated Tet addition. Similarly, *Flavobacterium* (family Flavobacteriaceae, 0.37%) and *Clostridium sensu stricto* (family Clostridiaceae, 0.41%) exhibited increased abundances at 2 mg·L^−1^ and 5 mg·L^−1^ accumulated Tet addition.

##### Root Sample Analysis

In root samples (with R-CK1 and R-CK2 combined as the CK and R-Tet1 and R-Tet2 representing accumulated Tet addition of 2 and 5 mg·L^−1^, respectively), Tet exposure significantly enriched resistant taxa (Figure 8). The rhizospheric microbial communities under Tet stress were primarily characterized by the proliferation of antibiotic-resistant or stress-tolerant taxa and a decline in sensitive taxa. At 2 mg·L^−1^ and 5 mg·L^−1^ Tet accumulation addition, OTUs associated with Rhizobiaceae (0.34%) and Comamonadaceae (0.37%) showed notable increases in abundance, indicating their dominance under Tet stress. In contrast, the rhizospheric microbial community in the CK exhibited greater diversity and stability, with no significant enrichment of specific taxa. As the accumulated Tet addition increased to 2 mg·L^−1^, Rhizobiaceae (0.34%), Comamonadaceae (0.37%), and Chitinophaga-ceae (0.38%) exhibited significant enrichment in some samples, while sensitive taxa, such as certain members of Verrucomicrobiota (8.25%) and Planctomycetota (5.42%), showed significant declines or even elimination. With further increases in accumulated Tet addition to 5 mg·L^−1^, these resistant taxa became more abundant, forming dominant communities with ecological advantages in the rhizosphere. However, at 10 mg·L^−1^ accumulated Tet addition, the root system of *E. crassipes* began to decay.

In summary, the rhizospheric microbial community evolved into a resistant and stress-tolerant dominant community, primarily comprising Rhizobiaceae (0.34%), Co-mamonadaceae (0.37%), and Chitinophagaceae (0.38%) under low to moderate Tet stress (2 to 5 mg·L^−1^). *E. crassipes* apparently maintained a relatively balanced rhizospheric environment, with enriched resistant taxa likely contributing to maintaining normal plant growth with a moderate Tet addition (5 mg·L^−1^). However, with the deterioration of plant health, the rhizospheric environment became imbalanced with higher Tet addition (10 mg·L^−1^).

### 3.3. Correlation Analysis of Microbial Taxa and Environmental Factors

**Figure 9:**
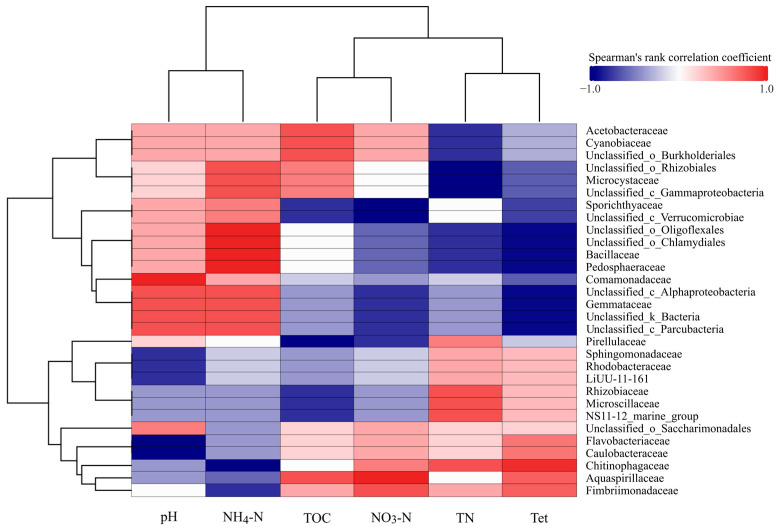
Correlation heatmap of microbial taxa and environmental factors in the rhizosphere. The vertical axis represents the top 30 families in terms of abundance; the horizontal axis represents the environmental factor indicators of correlation analysis, namely, pH, NH_4_^+^-N, TN, NO_3_^−^-N, TOC, and Tet.

To further explore microbial responses to environmental factors, correlations between family abundance and key environmental variables were analyzed (Figure 9). The heatmap highlights relationships between the top 30 families and environmental factors, namely, pH, NH_4_^+^-N, TN, NO_3_^−^-N, TOC, and Tet.

A detailed analysis revealed that families exhibited significant positive correlations with Tet, predominantly the families Chitinophagaceae (2.89%, ρ = 0.95), Flavobacteriaceae (0.48%, ρ = 0.63), Caulobacteraceae (0.70%, ρ = 0.63), Aquaspirillaceae (0.13%, ρ = 0.74), and Fimbriimonadaceae (0.31%, ρ = 0.74) (ρ denotes the Spearman’s rank correlation coefficient, where ρ ranges from −1 to +1, indicating the strength and direction of the relationship between variables). The Chitinophagaceae family (2.89%) demonstrated strong positive correlations with total nitrogen (TN) (ρ = 0.8) and nitrate nitrogen (NO_3_^−^-N) (ρ = 0.6) alongside a negative correlation with ammonium nitrogen (NH_4_^+^-N) (ρ = −1). The Rhizobiaceae (1.86%) and Microscillaceae (1.20%) families showed strong positive correlations with TN (ρ = 0.8) while exhibiting significant negative correlations with total organic carbon (TOC) (ρ = −0.8).

## 4. Discussion

Tet exposure significantly altered the rhizospheric environment of *E. crassipes*, impairing plant growth, nutrient cycling, and microbial community composition, suggested that Tet stress significantly inhibited the growth of *E. crassipes* [40]. A significant drop in pH value with an accumulated Tet concentration of 10 mg·L^−1^ could be caused by the decay of *E. crassipes* roots, resulting in the release of large amounts of organic acids, which likely contributed to the acidification of the rhizospheric environment [41]. The amount of accumulated Tet from 2 mg·L^−1^ to 5 mg·L^−1^ led to the decrease in NH_4_^+^-N, NO_3_^−^-N, and TN levels, likely due to nitrifying and denitrifying bacteria inhibition [42,43], while higher accumulated Tet from 5 mg·L^−1^ to 10 mg·L^−1^ increased these nitrogen forms, possibly due to the release of organic nitrogen by root decay [44]. A significant increase in TOC under Tet stress indicated changes in organic carbon dynamics driven by antibiotic effects on microbial activity [45]. These findings highlight the complex impacts of antibiotic contamination on plant–microbe interactions and nutrient cycling.

The present study also revealed a significant increase in both the types and total amounts of LMWOAs secreted by *E. crassipes* roots under Tet stress. The alteration of root exudation patterns under Tet-induced stress suggested that LMWOA secretion could serve as a protective mechanism against Tet toxicity or aid in maintaining rhizospheric stability [46]. Organic acids are known to modulate rhizospheric pH and influence microbial interactions [47,48]. Specifically, the increased concentration of tartaric acid may buffer against oxidative stress or support metabolic adjustments under Tet-induced stress [49]. Furthermore, the high concentration of acetic acid aligns with its previously reported role as a signaling molecule that regulates plant stress responses and rhizospheric microbial activity [50]. The results in this study indicated the critical role of organic acids in the plant’s adaptation to antibiotic stress and provided insights into their functional contributions to rhizospheric stability under contaminated conditions.

The restructuring of microbial communities exposed to Tet was evident in both aquatic and rhizospheric environments. Proteobacteria (46.6%) consistently dominated across all samples, reflecting its ecological resilience and biogeochemical significance [51]. Secondary phyla such as Bacteroidota (8.25%) and Actinobacteriota (2.69%) showed environment-specific responses, with notable enrichment in Tet treatment samples. These shifts highlight the selective pressure imposed by Tet stress, promoting antibiotic-resistant and degradative taxa, while suppressing sensitive groups such as Verrucomicrobiota (8.25%) and Planctomycetota (5.42%) [52].

In aquatic environments, microbial communities exhibit greater diversity and variability due to dynamic abiotic factors such as oxygen levels and nutrient availability [53]. Tet amplifies these dynamics, enriching resistant taxa like Bacteroidota without compromising overall diversity [54]. The richness of Rhodobacteraceae (genus *Rhodobacter*, 3.71%) under Tet stress highlights its metabolic versatility in organic pollutant degradation and antibiotic resistance [55,56], while *Flavobacterium* (family Flavobacteriaceae, 0.37%) and *Clostridium sensu stricto* (family Clostridiaceae, 0.41%), associated with organic matter decomposition and antibiotic degradation, underline the adaptive capacity of resistant microbial populations in mitigating Tet pollution in aquatic environments [57]. In contrast, rhizospheric communities are influenced by both chemical stress and root-associated environmental factors. The enrichment of Actinobacteriota (2.69%) and Bacilliota (2.47%) under Tet stress underscores the rhizosphere’s role as a selective niche for antibiotic-resistant taxa [58].

In the present study, key rhizospheric taxa, including Rhizobiaceae (0.34%), Coma-monadaceae (0.37%), and Chitinophagaceae (0.38%), exhibited significant enrichment with low to moderate accumulated Tet concentrations (2 to 5 mg·L^−1^). Rhizobiaceae (0.34%), critical for nitrogen cycling and plant–microbe interactions, increased with the rise in Tet levels, suggesting a tolerance mechanism that enhances rhizospheric stability. Similarly, Comamonadaceae (0.37%), known for its metabolic versatility in degrading organic pollutants, gained ecological advantages under Tet stress by metabolizing antibiotics or degradation intermediates [59]. Beyond their known Tet resistance via efflux pumps (e.g., TetA/C families) [60], genomic evidence suggested that this taxon may employ β-oxidation pathways (upregulated under carbapenem stress in related Comamonas strains) to metabolize antibiotic-derived toxic intermediates, while biofilm formation (supported by floc-associated growth in denitrifying sludge) could synergistically reduce intracellular drug accumulation [61]. Additionally, their genomic islands carrying Class 1 integrons (e.g., *blaGES-5*) and IncP-1 plasmids likely facilitate horizontal acquisition of resistance determinants [61]. Chitinophagaceae (0.38%), nearly absent in the CK, was selectively enriched under Tet stress. This taxon, associated with complex organic matter degradation, likely occupied a functional niche in the rhizosphere by metabolizing antibiotic degradation products or other organic resources. Certain members of Chitinophagaceae (0.38%) carried antibiotic resistance genes (ARGs), which further support their survival in antibiotic-contaminated environments [62].

To investigate the intrinsic mechanisms of microbial communities, correlation analyses between environmental factors and bacterial taxa at the family level were performed. By focusing on the 30 most abundant bacterial families, several notable associations were revealed. For instance, the Chitinophagaceae family (2.89%) demonstrates a remarkable adaptability to antibiotic-stressed environments through evolved resistance mechanisms, including efflux pump systems and enzymatic degradation pathways (e.g., aromatic ring-cleaving enzymes) that mediate antibiotic detoxification [54,55]. This ecological competence likely underpins their significant enrichment under Tet selection pressure, as evidenced by the strong positive correlation with Tet concentrations (ρ = 0.95). Notably, our correlation analysis revealed dual nutrient interactions: (1) positive associations with total nitrogen (TN, ρ = 0.80) and nitrate nitrogen (NO_3_^−^-N, ρ = 0.60), aligning with previous findings on their nitrogen-cycling potential in aquatic ecosystems [54]; (2) a perfect negative correlation with ammonium nitrogen (NH_4_^+^-N, ρ = −1.00), suggesting either ammonium sensitivity or competitive exclusion by specialized ammonium-utilizing consortia. These contrasting nitrogen responses highlight the metabolic versatility of Chitinophagaceae (2.89%) in redox-variable environments [63,64]. The Rhizobiaceae (1.86%) and Microscil-laceae (1.20%) families exhibit a synergistic correlation in the heatmap, showing significant positive correlation with total nitrogen (TN, ρ = 0.8) and negative correlation with total organic carbon (TOC, ρ = −0.8). This pattern reflects their complementary metabolic strategies under nitrogen-enriched and organic-carbon-limited conditions [65,66]. Rhizobiaceae, encompassing nitrogen-fixing symbionts like *Bradyrhizobium* spp., thrives in nitrogen-rich environments through root nodule associations [65]. Their TN-positive correlation aligns with their ecological role in nitrogen transformation, particularly under autotrophic conditions. Conversely, negative correlation between Microscillaceae and TOC was explained by its mixotrophic metabolism under inorganic carbon supplementation conditions [66], where it combines inorganic carbon fixation via upregulated Calvin–Benson cycle enzymes (RuBisCo, PGK, GAPDH) with hydrogen-driven denitrification. This dual strategy sustains nitrogen removal capacity through enhanced enzymatic activities (NAR, NIR, and NOR) in low-TOC environments [66]. Their functional synergy likely stems from niche partitioning. Rhizobiaceae enhances nitrogen availability through fixation [65], while Microscillaceae exploits inorganic carbon to drive denitrification [66]. This metabolic complementarity was further supported by improved electron transfer capacity under inorganic carbon conditions [66], facilitating cross-species interactions in organic-carbon-depleted systems.

## 5. Conclusions

Tet stress significantly inhibited the growth of *E. crassipes*, decreased the pH value in rhizospheric water, changed the organic carbon dynamics and nitrogen cycling in the aquatic system, and stimulated plant roots to secrete abundant LMWOAs, mainly tartaric acid and acetic acid, to resist antibiotic stress. Additionally, Tet exposure altered rhizospheric microbial diversity and community structure, favoring resistant taxa such as Rhizobiaceae (0.34%), Comamonadaceae (0.37%), and Chitinophagaceae (0.38%), which play key roles in nutrient cycling and pollutant degradation. At a moderate accumulated Tet concentration from 2 to 5 mg·L^−1^, microbial communities exhibited adaptive responses, suggesting their potential for bioremediation. In the aquatic environment, Tet promoted adaptive taxa like Bacteroidota (8.25%) and Actinobacteriota (2.69%), while in the rhizosphere, root exudates enriched antibiotic-degrading microbes and suppressed sensitive taxa. Tet exposure also shifted microbial metabolic pathways, notably increasing the secretion of acetic acid and tartaric acid. These findings underscore the potential cascading ecological effects of Tet contamination, emphasizing the need for careful antibiotic management. Further studies could concern the molecular mechanisms of Tet degradation and the interactions between plant roots and microbial communities to enhance bioremediation strategies. Nevertheless, it should be noted that this experiment was conducted under controlled laboratory conditions, which may not fully conform to the complexities of natural aquatic ecosystems. In addition, the highest Tet concentration (10 mg·L^−1^) induced visible root decay, influencing microbial responses under extreme stress. The relatively short duration of the present study may restrict the under-standing of the long-term ecological impacts and recovery dynamics of the aquatic system under antibiotic exposure conditions. Therefore, in situ field studies, expanded ranges of Tet concentrations, multi-omics approaches for comprehensive functional analysis, and extended observation periods will become the important directions in future studies.

## Data Availability

The data presented in this study are openly available in Zenodo at https://doi.org/10.5281/zenodo.14946356 (accessed on 28 February 2025), reference number 14946356.

## Abbreviations

The following abbreviations are used in this manuscript:

Tet	Tetracycline
NH_4_^+^-N	Ammonium nitrogen
NO_3_^−^-N	Nitrate nitrogen
TN	Total nitrogen
TOC	Total organic carbon
LMWOAs	Low-molecular-weight organic acids
ARGs	Antibiotic resistance genes
OTUS	Operational taxonomic units
